# Present Scenario of Long Non-Coding RNAs in Plants

**DOI:** 10.3390/ncrna3020016

**Published:** 2017-03-24

**Authors:** Garima Bhatia, Neetu Goyal, Shailesh Sharma, Santosh Kumar Upadhyay, Kashmir Singh

**Affiliations:** 1 Department of Biotechnology, BMS Block I, Panjab University, Sector 25, Chandigarh-160014, India; garibhat@gmail.com (G.B.); neetu12goyal@gmail.com (N.G.); 2 National Agri-Food Biotechnology Institute, C-127, Industrial Area, S.A.S. Nagar, Phase 8, Mohali 160071, Punjab, India; haitoshailesh@gmail.com; 3 Department of Botany, Panjab University, Chandigarh 160014, India; santoshnbri@hotmail.com

**Keywords:** non-coding RNA, lncRNAs, genome-wide identification, biological roles, lncRNA databases

## Abstract

Small non-coding RNAs have been extensively studied in plants over the last decade. In contrast, genome-wide identification of plant long non-coding RNAs (lncRNAs) has recently gained momentum. LncRNAs are now being recognized as important players in gene regulation, and their potent regulatory roles are being studied comprehensively in eukaryotes. LncRNAs were first reported in humans in 1992. Since then, research in animals, particularly in humans, has rapidly progressed, and a vast amount of data has been generated, collected, and organized using computational approaches. Additionally, numerous studies have been conducted to understand the roles of these long RNA species in several diseases. However, the status of lncRNA investigation in plants lags behind that in animals (especially humans). Efforts are being made in this direction using computational tools and high-throughput sequencing technologies, such as the lncRNA microarray technique, RNA-sequencing (RNA-seq), RNA capture sequencing, (RNA CaptureSeq), etc. Given the current scenario, significant amounts of data have been produced regarding plant lncRNAs, and this amount is likely to increase in the subsequent years. In this review we have documented brief information about lncRNAs and their status of research in plants, along with the plant-specific resources/databases for information retrieval on lncRNAs.

## 1. Introduction

Studies in the recent past have highlighted the pervasive nature of eukaryotic transcription and, hence, have strongly emphasized on the complexity involved in the expression of eukaryotic genomes [1]. For instance, it has been reported that approximately three-fourths of the human genome undergoes transcription [2]. In *Saccharomyces cerevisiae*, up to 85% of the genome is expressed [3]. Likewise, approximately 82% of the annotated genome has been reported to be transcribed in *Oryza sativa* (rice), a staple crop plant [4]. Eukaryotic genomes are not simple and ordered substrates of transcription. The notion that transcriptomes are derived solely from protein-coding and some specific non-coding RNA genes (such as small nuclear RNAs [snRNAs], small nucleolar RNAs [snoRNAs], transfer RNAs [tRNAs], or ribosomal RNAs [rRNAs]) is obsolete [1,5]. In fact, numerous studies indicated that RNA polymerase II could be present at nearly any genomic location [6,7], which drew the attention of researchers to an entire spectrum of RNA molecules beyond the traditionally known ones.

The presence of untranslated RNAs has been acknowledged for a considerable time now and have been associated with transcriptional and translational regulation, RNA modification, epigenetic modification of chromatin structures, etc. [8,9,10]. These untranslated RNA molecules are known by various names; for instance, the phrases “small RNAs” (sRNAs) and “non-coding RNAs” (ncRNAs) are generally used in the context of bacteria and eukaryotes, respectively. At various earlier instances, these RNAs have also been referred to as “non-protein coding RNAs” (npcRNAs) [11].

As per the popular definition, a transcript without an open reading frame (ORF) is referred to as ncRNA, particularly, when no experimental validation or phylogenetic evidence is available for any ORF [5]. In a broad sense, these are categorized as housekeeping ncRNAs and regulatory ncRNAs. The former are usually expressed constitutively and include rRNAs, tRNAs, snRNAs, and snoRNAs. The regulatory ncRNAs can further be categorized as (1) small RNAs that include microRNAs (miRNAs), small interfering RNAs (siRNAs), and Piwi-interacting RNAs (piRNAs), and (2) long non-coding RNAs (lncRNAs) based on their length in nucleotides. Essentially, lncRNAs are described as ncRNA transcripts of lengths no more than 200 nucleotides. 

Like protein-coding messenger RNAs (mRNAs), the majority of lncRNAs in eukaryotes are transcribed by RNA polymerase II. These are regarded as stable “typical” or classic lncRNAs [12]. Nevertheless, some lncRNAs are transcribed by RNA polymerase III, which was previously considered to form only infrastructure RNAs like tRNA and 5S rRNA [13]. In plants, lncRNAs are additionally transcribed by RNA polymerase IV and V [14,15]. The lncRNAs transcribed by plant-specific RNA polymerase V are involved in the process of RNA-directed DNA methylation (RdDM); hence, representing a different class of regulatory lncRNAs. In the model plant *Arabidopsis thaliana*, such class of lncRNAs is anticipated to act as a scaffold. The small RNAs (siRNAs) that are incorporated into Argonaute (binding to chromatin), base pair with this class of lncRNAs; hence, it facilitates the recruitment of Argonaute to the definite genomic loci [16,17]. Unlike the mRNAs, lncRNAs are primarily located within the nucleus [18]. These are generally found in very low quantity and in specific patterns in various tissue types [19]. These are also identified and classified on the basis of polyadenylation. Compared to the polyadenylated lncRNAs, non-polyadenylated lncRNAs are shorter in length [20]. Recently, non-polyadenylated lncRNAs have been identified in response to numerous stress conditions in *A. thaliana* [21].

## 2. Salient Features/Characteristics of lncRNAs

### 2.1. Biotypes/Classes

The lncRNAs biotypes have been defined on the basis of their genomic locations in relation to the neighboring genes. The broad classes are as follows: (1) intergenic, when lncRNA is present within the genomic interval between two genes; (2) intronic, when it is derived from an intron; (3) sense, when it overlaps the exons on the same strand; (4) antisense, when it overlaps the exons on the opposite strand; and (5) bidirectional, when the expression of lncRNA and an adjacent coding transcript on the opposite strand is initiated in close genomic proximity [5].

With the aid of biotype information, a researcher can focus on a particular subset of putative lncRNAs predicted in silico. Based on the reference annotation from the ENCODE (ENCyclopedia Of DNA Elements) project, GENCODE v7 (a project that has generated reference gene annotation and experimental confirmation for human and mouse genomes) introduced 12 biotypes of lncRNAs, which have been compiled in different databases for human lncRNAs and are mainly used to filter human lncRNA transcripts [22,23].

### 2.2. Similarity to mRNAs

Although lncRNAs in general lack protein-coding ability, these are similar to mRNAs in several ways [24] like RNA polymerases responsible for lncRNA transcription (as discussed above), polyadenylation, 5′ capping, and alternative splicing patterns [22]. The majority of lncRNAs are spliced with exon/intron lengths similar to that of mRNA coding genes [25,26]. However, exceptions have been observed in plants, such as *Cicer arietinum* (chickpea) and *Gossypium arboretum* (cotton), in which the mean exon length of long intergenic ncRNAs (lincRNAs) was reported to be substantially higher than that of mRNAs [27,28].

### 2.3. Tissue-Specificity

Tissue-specific expression levels of lncRNAs have been widely observed in mammals [29,30]. Such observations indicate the plausible role these transcripts play in differentiation, development, repair, maintenance, and various other processes. Recent studies in plants have also revealed tissue-specific lncRNAs expressed at particular developmental stages. The results suggest that lncRNAs are involved in fiber development in *G. arboretum* [28], flower development in *C. arietinum* [27], flower and fruit development in *Fragaria vesca* (woodland strawberry) [31], and floral organ and root development in *Morus notabilis* (mulberry) [32].

### 2.4. Cell-Type-Specificity

In the studies revealing tissue-specificity of lncRNAs, a general trend of lower expression levels has been observed for lncRNAs as compared to the protein-coding genes. This could be attributed to the consistently low levels of lncRNAs in all the cells, or expression in only a few specific cells/cell sub-populations [33,34]. Cell-type-specific lncRNAs have been widely identified in animals and humans. For example, discrete and abundant expression of lncRNAs was observed in the developing human neocortex based on single-cell transcriptomics. Despite their low abundance in tissues, lncRNAs, *LOC646329*, were found to be enriched in single radial glia subpopulation [34]. In a recent study on expression map of *Arabidopsis* roots, cell-type-specific lincRNAs were identified; however, lower expression levels of lincRNAs were observed in comparison to mRNAs even in specialized cell types [35].

### 2.5. Mechanisms of Action

Several compelling clues have been obtained in the last decade to reveal the functions of RNA beyond its conventional capacity as a messenger for protein-coding genes [36]. Studies by many research groups have revealed that lncRNAs are involved in epigenetic modification-dependent pre-transcriptional regulation, transcriptional regulation, and post-transcriptional regulation. Additionally, they act as scaffolds, endogenous target mimics of miRNAs, and precursors of ncRNAs. Since several exclusive reviews on current perspectives of molecular mechanisms and functions of lncRNAs are available, we suggest referring to the articles by Ponting et al., Zhu and Wang, Kung et al., Marín-Béjar and Huarte, and Wu et al. for in-depth description [5,37,38,39,40].

## 3. Emerging Significance of lncRNAs and Status in Plants

In a pioneering study conducted in 1992, Lukiw et al. first reported BC200 RNA, which were polyadenylated 200-nucleotides primate brain-specific lncRNAs [41]. Nearly 25 years later, a plethora of lncRNAs has been identified across diverse organisms. Concomitantly, the efforts have been made towards understanding the biological mechanisms of these transcripts. This is reflected by the augmented cognizance of involvement of lncRNAs in numerous molecular and regulatory processes [42]. Though several advances have been made in respect to the identification processes (like differentiating between protein-coding and ncRNAs) and functional characterization of lncRNAs, it is presumed that only the tip of an iceberg has been explored until now. Numerous efforts are being made in this direction using bioinformatics aids, computational tools, and high-throughput sequencing technologies, such as the lncRNA microarray technique, RNA-sequencing (RNA-seq), RNA capture sequencing, (RNA CaptureSeq), etc. [40]. Currently, more than half a million lncRNA transcripts have been identified across different eukaryotes with leading research in humans and mice. In fact, several new web-based tools and well-established databases are available to identify, study, and compare lncRNAs in humans and understand the roles of these long RNA species in several diseases, for example, LNCipedia [43], lncRNome [44], Co-lncRNA [45], starBase v2.0 [46], lncRNADisease [47], lnc2Cancer [48], etc. For comparative evaluation of these emerging tools, please refer to reviews by Fritah et al., Jalali et al., and Yotsukura et al. [23,49,50].

Interestingly, *GmENOD40*, a *Glycine max* (soy bean) lncRNA gene of 700 nucleotides was reported by Yang et al. in 1993 [51]. However, over the decades, the progress of lncRNA research lagged behind as compared to that in humans and other mammals. Nevertheless, initially triggered by full-length cDNA cloning and tiling microarrays, the discovery of plant lncRNAs is, at present, primarily stimulated by next-generation RNA-seq.

The initially reported biologically-important plant lncRNA genes such as *GmENOD40* [51], *MtENOD40* [52], *TPS11* [53], *OsPI1* [54] etc. and the recently reported plant lncRNA genes such as *AtIPS1* [55], *COOLAIR* [56], *COLDAIR* [57], *LDMAR* [58,59], etc. have provided insights into the diverse biological roles played by these long RNA species. For example, *ENOD40* is expressed during nodule organogenesis in plants like *G. max*, *Medicago truncatula*, *Medicago sativa*, etc. In *M. truncatula*, *MtENOD40* RNAs were identified as a distinct class of untranslated RNAs (referred to as “riboregulators”) localized in the cytoplasm of cells in the nodule primordium, which affect the growth control and differentiation [52]. Another lncRNA, *AtIPS1* [55], was studied in *A. thaliana* and was found to interact with miRNA ath-miR399. The interaction involved an interrupted pairing between the lncRNA and miRNA at the expected miRNA cleavage site (that is, a three-nucleotide bulge was present between the tenth and eleventh positions at the 5′ end of the miRNA). Hence, *AtIPS1* was identified as a target mimic of ath-miR399 due to its inability to be cleaved by the miRNA and in turn sequestering it. Owing to this mechanism of target mimicry, the actual target of ath-miR399, that is *PHO2* mRNA, tends to accumulate when *AtIPS1* is over expressed. Likewise, several plant molecular functions and biological processes have been found to be driven by lncRNAs; for instance: vernalization, fertility, photomorphogenesis, protein re-localization, phosphate homeostasis, alternative splicing, modulation of chromatin loop dynamics, etc. Our present understanding of these functions in plants with respect to the lncRNAs has been discussed in detail in some recent review studies conducted by Zhu and Wang [37], Kim and Sung, Zhang et al., Liu, J et al., and Liu, X et al. [60,61,62,63]. 

In addition to these lncRNA genes, significant amount of data with respect to plant lncRNAs have been generated owing to the recent widespread application of high-throughput RNA-seq and computational pipelines. This upsurge in the amount of publicly available RNA-seq data is not only facilitating global identification and in silico characterization of lncRNAs in diverse plant species, but is also paving the way for understanding expression patterns and potential functions of lncRNAs. 

Table 1 provides an overview of the recent studies that have been conducted for genome-wide/global identification and characterization of plant lncRNAs primarily based on transcriptome analysis (using high-throughput next generation sequencing strategies and computational pipelines). Many studies have revealed thousands of putative lncRNAs being expressed in plants in specific tissues, at particular developmental stages, or in response to stress conditions.

## 4. Managing the Information: Repositories/Databases of Plant lncRNAs

As reflected by Table 1, a significant amount of data has been produced regarding plant lncRNAs. The growing availability of computational aids and standardization of pipelines to conduct in silico identification and characterization of lncRNAs, as well as the development of new high-throughput technologies are likely to catalyze the pace of plant lncRNA research, which in turn would generate more data. Therefore, it is only reasonable to create, generate, manage, improve, and/or update repositories for organizing plant lncRNA information. Databases or web-based platforms that blend inclusive information about lncRNAs can enhance our comprehension of several biological processes. The established comprehensive databases available exclusively for human and mammal lncRNAs (in addition to basic information) include details of functional annotation, tissue expression, epigenetic factors, single nucleotide polymorphisms, disease associations, phylogenetic conservation, and interactions of lncRNAs with other RNAs and proteins. Comparatively, plant-specific lncRNA databases are not that comprehensive. In the subsequent section, we have reviewed the currently available databases for managing, depositing, and understanding plant lncRNAs. Some recently released and updated databases are apparently quite promising in terms of efficient management and retrieval of significant information. 

An overview of the currently available databases with entries from plants has been presented in Table 2.

Further, each of these databases has been summarized as follows (commencing with the more comprehensive repositories and continuing to the more plant-specific ones):

### 4.1. NONCODE v4

NONCODE is an elaborated database for eukaryotic non-coding RNAs with the exception of tRNAs and rRNAs [94]. The updated version of the database, NONCODE v4, was released two years after the launch of NONCODE v3 owing to the rapid increase in the number of lncRNAs identified in various organisms. It includes information for 16 wide-ranging species (for instance, *Homo sapiens*, *Mus musculus*, *Gorilla gorilla*, *Drosophila melanogaster*, *A. thaliana*, etc.). The basic information of lncRNAs such as location, strand, length, exon number, class (or biotype), isoforms, coding potential, and sequence is available in addition to the advanced information (for some species such as *H. sapiens* and *M. musculus*) such as the expression profile across various tissues, conservation, disease relation, and related literature. 

NONCODE includes data from three types of sources primarily: GenBank, specialized databases, and the literature. In NONCODE v4, additional data were collected from studies published since the last update and the latest versions of several public databases such as Ensembl 2015 [102], Refseq—updated mammalian reference sequences [103], lncRNAdb v2.0—reference database for functional lncRNAs [36], and GENCODE v7—catalog of human lncRNAs [22]. Furthermore, new online services have been introduced to fill the previously existing lacunae such as the lack of directives for novel lncRNA discovery. iLncRNA, an online lncRNA-identification pipeline is one such improvement, which is based on the assembled transcript data supplied by the user in the format of gff or gtf files. With the consent of the user, the predicted results could be accumulated in NONCODE per se. Another improvement is an ncRNA ID conversion tool, which facilitates conversion of RefSeq or Ensembl ID into NONCODE ID; hence, it enables users to query accessions from diverse RNA databases. 

The interface of the database is user-friendly, and options like browse, search, and download are conveniently accessible to a user. Moreover, NONCODE has been included into other ncRNA repositories such as Functional RNA Database or fRNAdb (which provides information about non-coding yet functional RNAs in multiple organisms) [104], GeneCards (which is a non-redundant compendium of human ncRNA genes) [105], and DIANA-LncBase (which is an extensive database of interactions between miRNAs and lncRNAs in various human and mouse tissues) [106].

In short, NONCODE is an integrated knowledge database, which comprises comprehensive collection and annotation of lncRNAs. However, it does not specifically focus on plant lncRNAs. In fact, only 3853 lncRNA transcripts and 2477 lncRNA genes account for *A. thaliana* out of a huge collection of 527,336 and 337,880 lncRNA transcripts and genes in the database, respectively [107]. Interestingly, this indicates that merely 0.7% (approximately) of the total lncRNAs in NONCODE represent the only plant species included in the database, that is, *A. thaliana*.

### 4.2. lncRNAdb v2.0

Like NONCODE, lncRNAdb is a comprehensive repository of eukaryotic lncRNAs [36]. However, its distinguishing characteristic is that entries into lncRNAdb are manually curated from literature-supported evidence. In fact, lncRNAdb adopts strict criteria to include only those lncRNAs for which functional characterization has been conducted using knockdown or over-expression experiments. Owing to these characteristics, lncRNAdb is a reliable source of biologically-investigated lncRNAs and has been incorporated into other integrative databases like NONCODE and RNAcentral [108].

First published in 2011, the latest updated version of the database (LncRNAdb v2.0) was released in 2015. Currently, lncRNAdb v2.0 comprises nearly 283 entries spanning 71 different organisms. The information is supported by 921 references and 260 nucleotide sequences. Additionally, new features have been introduced. For instance, users with a potential lncRNA sequence can avail the lncRNAdb blast search to compare their sequence to any known functional lncRNA. Further, the information for all the results can be downloaded as an XML file or printer-friendly summary. In fact, the entire database can be downloaded using the representational state transfer (REST) application program interface (API). REST API has improved data accessibility by enabling the users to download raw data files programmatically. Data can be retrieved with flexibility, that is, specific content can be obtained for individual lncRNA (like COOLAIR, COLDAIR, or HOTAIR) or for multiple entries at once. Additional information with apt examples is available under “Tools and Features” link on the database website [109]. Not compromising on the integrity of the nucleotide sequence data, the database provides corresponding International Nucleotide Sequence Database Collaboration (INSDC) IDs and links out sequences to the European Nucleotide Archive (ENA).

Furthermore, with the new user-friendly interface, one can easily access the profile of an entry that describes the genomic characteristics, expression, function, and other relevant information. For *H. sapiens* lncRNA entries with the corresponding Ensembl Gene ID, expression data for 16 human tissues are available from the Illumina Body Atlas [110].

Despite the striking merits of lncRNAdb v2.0, its applicability is apparently limited for a biologist studying lncRNAs in plants. Most (~75%) of the catalogued lncRNAs in the database are from mammals. On searching for all the entries from *A. thaliana*, only seven results were obtained. Likewise, for other plant species such as *O. sativa*, *M. truncatula*, *Brassica rapa*, *G. max*, etc., few entries were observed (that is, less than 10).

### 4.3. RNAcentral

Unlike the two above discussed databases, RNAcentral aggregates data of all ncRNA types from all organisms, that is, including both prokaryotic and eukaryotic ncRNAs [100]. 

The ncRNA sequence data that are aggregated into RNAcentral are supported by an international consortium of RNA resources referred to as Expert Databases. Post its release in 2014, RNAcentral has collaborated with 12 new specialized ncRNA resources. The Arabidopsis Information Resource (TAIR) is one of the newly-integrated resources in the latest version (fifth release) of RNAcentral. Currently the RNAcentral Consortium comprises 40 Expert Databases, out of which 22 have been imported. Clearly, the approach has created a gateway for the users to access ncRNAs via single entry point. Data can primarily be accessed in three ways using the RNAcentral website: text search, sequence similarity search, and genome browser. Wherever applicable, the ncRNA sequences have been mapped to reference genomes from selected species; hence, sequences annotated with genomic coordinates can be viewed for specific species. Further, new species-specific identifiers have been provided to refer to unique RNA sequences for single species. 

The statistics available on the database website indicate that 209,384 lncRNA sequences are available. However, out of these, merely 670 and the lncRNAs are available for *Zea mays* and *A. thaliana*, respectively. 

The under-representation of plant lncRNAs in such comprehensive repositories reflects the infancy of research status of lncRNAs in plant species. Possibly, it also hints at the gap between genome-wide identification of plant lncRNAs and submission of the data to databases. Simultaneously, it highlights the need for more plant-specific lncRNA repositories, which can accommodate the plant lncRNAs reported recently in the literature. 

We now discuss the plant-specific databases that are currently available for ncRNA information and lncRNAs in particular:

### 4.4. TAIR10

The Arabidopsis Information Resource (TAIR) has been designed to provide comprehensive information in the form of genetic and molecular biology data for Arabidopsis [91]. The latest version of the resource, TAIR10, combines structure- and organization-related information about the Arabidopsis genome. Additionally, it takes into account details of the functions of its estimated 33,602 genes. 

In recent updates of the resource, fresh data from next-generation transcriptome sequencing (RNA-seq) were incorporated as evidence for gene model updates. 

Information about gene function and expression is based on experiments reported in the peer-reviewed literature, which is selected manually by TAIR curators. Microarray data are also available publicly at TAIR in both raw and analyzed forms. The primary source of such data is the Arabidopsis Functional Genomics Consortium (AFGC) cDNA arrays. The analyzed version of the microarray data from over 370 experiments can be viewed using hierarchical Java Tree Viewer.

TAIR is a relational database, which additionally provides access to web-based tools for querying and analyzing the stored data. To a user, TAIR serves as a central access point for Arabidopsis data; the sources of such data include large-scale sequencing and functional genomics projects, independent researchers, and the literature.

### 4.5. PlantNATsdb

Natural antisense transcripts (NATs) refer to the complementary transcripts of the protein-coding transcripts. These comprise a class of RNAs that include both protein-coding and non-coding transcripts [89]. As discussed earlier, antisense transcripts are one of the biotypes of lncRNAs, which are characterized by partial/complete overlapping with exons on the opposite strand. PlantNATsdb or plant NAT database is dedicated to serve as a reference database to investigate the regulatory function of NATs in the plant kingdom [92]. 

Approximately, 2138,498 NATs from 70 plant species have been included in the database by integrating various data sources such as TAIR9, Joint Genome Institue (JGI) Glyma1, JGI Cassava 1, etc. Additionally, GO (gene ontology) annotation and high-throughput small RNA sequencing data were incorporated to explore the biological function of NATs. The web interface of the database is user-friendly, interactive, and a graphical network browser is available that displays complex networks involving different NATs.

Furthermore, a GO annotation-based module—Gene Set Analysis—was designed to extract statistically significant GO categories that were overrepresented from the specific NAT network. The information in PlantNATsDB is freely available.

### 4.6. PLNlncRbase

PLNlncRbase is an easy-to-use resource that provides information exclusively for plant lncRNAs, particularly, those that have been identified experimentally [96]. In fact, it enables a user to browse through the repository based on diverse plant species (such as *A. thaliana*, *B. rapa*, *G. max*, *M. truncatula*, *Populus trichocarpa*, *Solanum lycopersicum*, *Triticum aestivum*, etc.) and/or lncRNA category (biotypes such as NATs, intergenic, intronic lncRNAs etc.). Currently, 1187 plant lncRNAs in 43 plant species have been manually curated from over 200 published studies. 

Detailed information can be retrieved for a specific entry including a lncRNA identifier, brief description of the potential biological role, sequence, biotype, an expression pattern of the lncRNA, tissue/developmental stage/condition for lncRNA expression, chosen method for studying lncRNA expression, PubMed ID (PMID) and/or digital object identifier (DOI) for referring to the original study, etc. Data can be freely downloaded from the database. Additional tools like Coding Potential Calculator (CPC), blast, etc. have been provided for further data analyses. 

Xuan et al., have reported that the database will be updated semimonthly [96]. If such a database continues to be regularly updated, it would promote future plant lncRNAs research. Moreover, this database provides a convenient submission interface for contribution of novel plant lncRNA entries by independent researchers. 

Undoubtedly, a database like PLNlncRbase is a strong step towards the establishment of a comprehensive and reliable plant lncRNA information source.

### 4.7. GreeNC

The Green Non-Coding (GreeNC) database comprises lncRNAs annotated in plants and algae [97]. Like PLNlncRbase, this database too exclusively provides information about lncRNAs identified across several plant species (that is, 37) such as *Ananas comosus*, *Arabidopsis lyrata*, *A. thaliana*, *Citrus sinensis*, *Malus domestica*, *Solanum tuberosum*, etc. However, the database comprises lncRNAs that have been annotated in silico based on reference transcripts, which were downloaded from Phytozome v10.3 [111] and by using highly specific and sensitive in-house bioinformatic pipelines. 

Presently, GreeNC database includes approximately 200,000 pages of information about more than 190,000 lncRNA transcripts from 37 plants and six algae. Out of these, 120,000 transcripts have been annotated as high-confidence lncRNAs. Further, 30% of these lncRNAs have been identified in *T. aestivum* and *Z. mays*.

All the sequences for each species can be downloaded in FASTA format at each species page. Besides sequences, information about the genomic coordinates, coding potential, GC content, and folding energy for all the identified lncRNAs can be accessed.

According to Gallart et al. [97] GreeNC database will be maintained properly, and annual updates will be conducted to improve the presently available genome annotations. Novel sequences identified from additional species will also be absorbed into the database. Furthermore, based on the RNA-seq data available in the public domain, additional information regarding lncRNA expression patterns will be incorporated. Eventually, the upgraded versions of the database will include details of phylogenetic conservation. 

Such a repository would prove to be a hub of plant lncRNAs identified in silico, which could possibly emerge as a comprehensive source of putative lncRNAs; however, subsequent experimental validation is necessary.

### 4.8. CANTATAdb

CANTATAdb is a simple and user-friendly database that comprises plant lncRNAs in 10 model plant species such as *A. thaliana*, *O. sativa*, *S. tuberosum*, etc. [98]. 

Like GreenC database, lncRNAs in CANTATAdb have been computationally identified with publicly available RNA-Seq sample data. The carefully evaluated and curated data in terms of expression levels, coding potential, and sequence alignments are freely available for searching, browsing, and downloading purposes.

A distinct feature of CANTATAdb is the annotation data, which includes predicted functions in context to lncRNA– miRNA interactions and/or splicing modulation. A user can easily search species-specific data based on useful filters for potential function, confidence level, coding potential, etc. In total, 45,117 lncRNAs have been included for the 10 plant species. Out of which, 11,896 lncRNAs have been assigned potential functions; of these, 440 have been considered to be involved in deregulation of miRNA functions, and 11,659 have been suggested to function as splicing modulators through masking splicing signals. 

CANTATAdb certainly draws the users a step forward in the direction of deciphering the potential regulatory functions of lncRNAs.

### 4.9. PNRD

As compared to the aforementioned plant-specific databases, plant ncRNA database (PNRD) is an integrated online platform to study different types of ncRNAs across various plant species [95]. In fact, it is an updated version of a plant miRNA database named PMRD (2010). The data sources include the literature and high-throughput sequencing data (both in-house data and that available in public repositories).

Currently, 25,739 entries of 11 different types of ncRNAs from 150 plant species are available in the database. However, information about lncRNAs is available for only four species, i.e., *A. thaliana*, *O. sativa*, *P. trichocarpa*, and *Z. mays*. 

With the aid of PNRD, users can calculate coding potential of sequences of interest using the CPC toolkit. For new miRNA discovery, an improved miRNA prediction toolkit has been provided. Moreover, two genome browsers are available for scanning ncRNA location along with coding genes and determining their relationship with epigenetic modifications. 

For a plant ncRNA researcher, PNRD could prove a useful resource and integrated platform for studying, searching, browsing, predicting, visualizing, and downloading different types of ncRNAs.

### 4.10. PLncRNAdb

A simple yet informative database, PLncRNAdb comprises more than 5000 lncRNAs collected from four plant species (*A. thaliana*, *A. lyrata*, *P. trichocarpa*, and *Z. mays*) [101].

A computational pipeline was built for lncRNA prediction in the plant species, and data were also collected from the literature. A striking feature of PLncRNAdb is the provision of relationships between lncRNAs and various RNA binding proteins (RBPs), which can be visualized as lncRNA-protein networks. The interactions between lncRNAs and RBPs have been predicted using the web server of catRAPID [112].

### 4.11. PLncDB

Plant long non-coding RNA database (PLncDB) was one of the initial attempts to provide information for a large number of plant lncRNAs collected from diverse resources [93]. However, till date, the database provides a comprehensive genomic view of *Arabidopsis* lncRNAs only.

On the basis of Reproducibility-based Tiling array Analysis Strategy (RepTAS) and RNA-seq data, more than 13,000 lncRNAs were found to be transcribed from intergenic regions of *A. thaliana* genome [66], which have been included in PLncDB. 

### 4.12. DsTRD

Danshen Transcriptional Resource Database (DsTRD) is a transcript resource dedicated to single plant species, *Salvia miltiorrhiza* (a medicinal model plant) [99]. As a comprehensive, yet plant-specific database, DsTRD includes information regarding the sequences and functional annotations of different types of transcripts, that is, protein-coding RNAs, lncRNAs, other ncRNA, miRNAs, and phasiRNAs.

It contains 76,531 transcribed sequences assembled from the RNA-seq data. Additionally, tissue expression for each transcript has been included, which was calculated and represented based on RNA-seq data. Moreover, information about RNAs associated with some pathways has also been provided. Databases like DsTRD could prove efficient tools to better investigate molecular processes for a particular plant under study. 

Briefly, the presently available databases act as both direct and indirect sources of information for studying plant lncRNAs. PLNlncRbase, GreeNC, and CANTATAdb are specialized databases, in which considerable information has been incorporated about lncRNAs across diverse plant species. While PLNlncRbase integrates experimentally identified lncRNAs, the other two databases include lncRNAs predicted in silico. Nevertheless, these are useful sources of information with respect to plant lncRNA research. Generalized information repositories like NONCODE v4, lncRNAdb v2.0, and RNAcentral enable a researcher to study ncRNAs in several eukaryotic and prokaryotic organisms; however, for exclusive studies on plant lncRNAs, these sources are likely to be insufficient. Resources such as TAIR10 act as an indirect source of lncRNA information, that is, distinctly classified lncRNAs are not available; however, the availability of abundant genomic information plus expression data can facilitate lncRNA studies in the model plant. For the analysis of various types of plant ncRNAs (for example, snoRNAs, snRNAs, rRNAs, etc.) in addition to lncRNAs, PNRD is a useful source. Likewise, PlantNATsdb provides a platform to study NATs in addition to lncNATs across several plant species. Simple databases, such as PLncRNA, PLncDB, and DsTRD offer lncRNA information specific to only a few plant species. Interestingly, most databases do not have a provision of submitting new data about plant lncRNAs. Currently, experimentally-verified new plant lncRNAs can be submitted online at PLNlncRbase and lncRNAdb v2.0.

## 5. Concluding Remarks

Owing to the increasing number of lncRNA studies in plants and consequently, the increasing volume of data, comprehensive resources dedicated towards plant lncRNAs are the utmost need of the hour. In future, development of lncRNA prediction tools (like those for animals and humans) based on data beyond sequences (like gene expression data and protein-interaction data) would further catalyze the identification process. Well-managed repositories will enable the researchers to draw functional significance for both novel and already known lncRNAs. However, the existing lncRNA resources have been unsuccessful to encompass most of the newly-identified lncRNAs in recent studies. Consequently, it is required that the existing databases are updated frequently and on a regular basis.

## Figures and Tables

**Table 1:** Studies showing genome-wide identification of long non-coding RNAs (lncRNAs) in plants over the last decade.

S.No.	Year	Publication Details and Reference No.	Approach of Identification	Plant Species	Biotypes and Number	Tissues/Developmental Stages	Stimuli/Biological Process		
							Abiotic Stress	Biotic Stress	Others
1.	2007	Wen et al. In silico Biology [64]	Expressed and genomic sequence data + Computational Pipeline	*Medicago truncatula* (Barrelclover)	mRNA-like non-coding transcripts: 503	-	✕	✕	✕
2.	2009	Amor et al. Genome Research [11]	Genome-wide bioinformatic analysis of full-length cDNA databases	*Arabidopsis*	Long non-protein coding RNAs: 76	Inflorescences, stems, and leaves	✓ Salt stress, Phosphate starvation, and Water stress	✕	✕
3.	2011	Xin et al. BMC Plant Biology [65]	Microarray Analysis + SBS Sequencing	*Triticum aestivum* (Wheat)	Long non-protein coding RNAs: 125	Leaf samples at 0 and 12 hours post inoculation	✓ Heat stress	✓ Powdery mildew infection	✕
4.	2012	Liu et al. The Plant Cell [66]	RNA sequencing + computational prediction	*Arabidopsis thaliana*	lincRNAs: 2708	Root and leaf samples of 30-day-old plants and two-week-old seedlings	✓ Drought, Cold, High-salt, and Abscisic acid treatment	✕	✕
5.	2012	Boerner and McGinnis PLoS ONE [67]	Full-length cDNA sequences + computational pipeline	*Zea mays* (Maize)	lncRNAs	-	✕	✕	✕
6.	2012	Lu et al. BMC Genomics [68]	Strand-specific RNA-seq + computational pipeline	*Oryza sativa* ssp. *japonica* cv. Nipponbare (Rice)	Cis-NATs: 3819	Seedlings and epidermal cells	✓ Drought, salt stress and cold treatment	✕	✕
7.	2013	Qi et al. Plant Mol. Biology [69]	Deep transcriptomic sequencing	*Setaria italic* (Foxtail millet)	lncRNAs: 584 ➢ lincRNAs: 494 ➢ lncNATs: 90	Shoots	✓ Drought	✕	✕
8.	2013	Wang et al. Plant Mol. Biology [70]	Deep RNA-seq	*Prunus persica* (Peach)	ncRNAs: 1417	Leaves, flowers, and fruits	✕	✕	✕
9.	2013	Yu et al. BMC Plant Biology [71]	RNA-seq + computational pipeline	*Brassica rapa*	Cis-NATs: 1031	Seedling (three weeks old) and inflorescence apices (two months old)	✕	✕	✕
10.	2014	Wang et al. Genome Research [72]	Strand-specific RNA-seq + strand-specific tiling arrays	*A. thaliana*	Sense–antisense transcript pairs: 37,238	Cotyledons, hypocotyls, and roots of seedlings	✕	✕	✓ Light
11.	2014	Zhu et al. New Phytologist [73]	Strand-specific RNA-seq	*A. thaliana*	lncRNAs	Two-week old seedlings	✕	✓ *Fusarium oxysporum*	✕
12.	2014	Li et al. Genome Biology [74]	EST databases, maize whole genome sequence annotation and RNA-seq datasets + computational pipeline	*Z. mays* (Maize)	lncRNAs: 20,163	Thirdteen distinct tissues (leaf, immature ear, immature tassel, seed, endosperm, embryo, embryo sac, anther, ovule, pollen, silk, and root and shoot apical meristem)	✕	✕	✕
13.	2014	Shuai et al. Journal of Experimental Botany [75]	RNA-seq + computational pipeline	*Populus trichocarpa* (Poplar)	lincRNAs: 2542	Mature leaves	✓ Drought	✕	✕
14.	2014	Zhang et al. Genome Biology [76]	Strand-specific RNA-seq + computational pipeline	*O. sativa* (Rice)	lncRNAs: 2224 ➢ lincRNAs: 1624 ➢ lncNATs: 600	Anthers, pistils, seeds five days after pollination, and shoots 14 days after germination	✕	✕	✓ Sexual reproduction
15.	2015	Chen et al. Planta [77]	High-throughput RNA-seq + computational pipeline	*Populus tomentosa* (Poplar)	lncRNAs: 1377	Tension, opposite, and normal wood xylem from 30-year old trees	✕	✕	✕
16.	2015	Hao et al. PLoS ONE [78]	RNA-seq data + computational pipeline	*Cucumis sativus* (Cucumber)	lincRNAs: 3274	Fruits at five ages, root, stem, leaf, male and female flowers, ovary, expanded fertilized and unfertilized ovary, base of the tendril, and tendril	✕	✕	✕
17.	2015	Zhu et al. Journal of Experimental Botany [79]	Paired-end strand-specific RNA-seq	*Solanum lycopersicum* cv. Ailsa Craig (Tomato)	lncRNAs: 3679	Fruits: immature green, mature green, breaker, pink, and red-ripe stages (Ripening)	✕	✕	✕
18.	2015	Wang et al. Scientific Reports [80]	Strand-specific paired-end RNA-seq + computational pipeline	*S. lycopersicum* (Tomato)	lncRNAs: 1565	Leaves	✕	✓ Tomato yellow leaf curl virus	✕
19.	2015	He et al. Frontiers in Plant Science [81]	RNA-seq + computational pipeline	*O. sativa* spp. Japonica cv. Nipponbare (Rice)	lncRNAs	Roots	✓ Cadmium stress	✕	✕
20.	2015	Wang et al. BMC Plant Biology [82]	High-throughput sequencing + bioinformatic analysis	*M. truncatula* (Barrelclover)	lncRNAs: 23,324	Leaves and roots	✓ Osmotic and salt stress	✕	✕
21.	2015	Kang and Liu BMC Genomics [31]	RNA-seq data + computational pipeline	*Fragaria vesca* (Woodland strawberry)	lncRNAs: 5884	Thirty-five distinct floral and fruit tissues and two vegetative tissues: seedlings and young leaves (Flower and fruit development)	✕	✕	✕
22.	2016	Zou et al., Science China Life Sciences [28]	Strand-specific RNA-seq + computational pipeline	*Gossypium arboretum* (Cotton)	lncRNAs: 5996 ➢ lincRNAs: 3510 ➢ lncNATs: 2486	Ovules and fibers on 1, 10, and 15 days post anthesis; leaves from 2-week-old seedlings (Fiber development)	✕	✕	✕
23.	2016	Tian et al., Journal of Experimental Botany [83]	RNA-seq + computational pipeline	*Populus*	lncRNAs: 7655	Leaves	✕	✕	✓ Hormone responses (Gibberellin)
24.	2016	Song et al. Genes [32]	RNA-seq data + computational pipeline	*Morus notabilis* (Mulberry)	lncRNAs: 1133	Winter bud, leaf, flower, root and bark	✕	✕	✕
25.	2016	Zhang et al. BMC Genomics [84]	RNA-seq + computational pipeline	*T. aestivum* (Wheat)	lincRNAs: 58,218	Leaves at 0, 1, 2, and 3 days post inoculation	✕	✓ Stripe rust and Powdery mildew infection	✕
26.	2016	Khemka et al. Scientific Reports [27]	RNA-seq data + computational pipeline	*Cicer arietinum* (Chickpea)	lincRNAs: 2248	Three vegetative tissues: germinating seedling, young leaves, and shoot apical meristem; and eight successive stages of flower tissues from closed flower bud to drooped flower (Flower development)	✕	✕	✕
27.	2016	Lv et al. BMC Genomics [85]	Ribosomal RNA depletion and ultra-deep total RNA-seq	*Zea mays* L. (Maize)	lncRNAs: 7245 ➢ lincRNAs: 6211 ➢ long intronic ncRNAs: 1034	Leaf	✓ Nitrogen stress	✕	✕
28.	2016	Chen et al. Mol Genet Genomics [86]	Genome-wide strategy	*Populus tomentosa* (Poplar)	lncRNAs: 388	-	✓ Nitrogen stress	✕	✕
29.	2016	Flórez-Zapata et al. BMC Genomics [87]	RNA-seq + computational pipeline	*Helianthus annus* (Sunflower)	lncRNAs: 25,327	Prophase I meiocytes from disc florets of the floral bud	✕	✕	✕
30.	2016	Joshi et al. PLoS ONE [88]	RNA-seq + computational pipeline	*Brassica napus* (Canola)	lncRNAs: 3181	Leaves	✕	✓ *Sclerotiniasclerotiorum* Infection	✕
31.	2016	Yuan et al. BMC Genomics [89]	Strand-specific RNA libraries + RNA-seq + computational pipeline	*A. thaliana*	lncRNAs: 1212	Shoot and root of 10-day-old seedlings	✓ Phosphate starvation	✕	✕
32.	2016	Kwenda et al. BMC Genomics [90]	Strand-specific RNA-seq + computational pipeline	*Solanum tuberosum* (Potato)	lincRNAs: 1113	Stems	✕	✓ *Pectobacterium carotovorum* subsp. *brasilience*	✕

Abbreviations used in Table 1: EST, expressed sequence tag; lncRNA, long non-coding RNA; lincRNA, long intergenic non-coding RNA; lncNAT, long non-coding natural antisense transcript; ncRNA, non-coding RNA; RNA-seq, RNA sequencing; SBS, sequencing by synthesis.

**Table 2:** An overview of the currently available databases for plant lncRNAs.

S.No.	Name of the Database	Publication Details and Reference No.	Plant Species	Number of Plant lncRNAs	Description/Main Features	Data Sources	Link/URL
1.	TAIR10	Lamesch et al., (2012) Nucleic Acids Research [91]	*Arabidopsis*	Information available about 33,602 genes of *Arabidopsis*	*Arabidopsis* gene structure and function annotation Plant-specific	AFGC cDNA arrays, the literature, and sequencing and function genomics projects	https://www.arabidopsis.org/
2.	PlantNATsDB	Chen et al. (2012) Nucleic Acids Research [92]	Seventy plant species	NATs (including both protein coding and non-coding transcripts): 2,146,803	A comprehensive database of NATs Plant-specific	Various data sources such as TAIR9, JGI Glyma1, JGI Cassava 1	http://bis.zju.edu.cn/pnatdb/
3.	PLncDB	Jin et al. (2013) Bioinformatics [93]	*Arabidopsis thaliana*	>13,000 lncRNAs	A comprehensive genomic database of *Arabidopsis* lncRNAs Plant-specific	Data in the study by Liu et al., 2012 [63]	http://chualab.rockefeller.edu/gbrowse2/homepage.html
4.	NONCODE v4	Xie et al. (2014) Nucleic Acids Research [94]	*A. thaliana*	3853 lncRNA transcripts and 2477 lncRNA genes	An integrated knowledge database with comprehensive collection and annotation of lncRNAs plus other ncRNAs Not plant-specific	The literature, specialized databases, and GenBank	www.bioinfo.org/NONCODEv4/
5.	PNRD	Yi et al. (2015) Nucleic Acids Research [95]	Four plant species: *A. thaliana*, *Oryza sativa*, *Populus trichocarpa*, and *Zea mays.*	5573 lncRNAs	A comprehensive integrated web resource for lncRNAs and other ncRNAs Plant-specific	Integration of data from other databases and publications	http://structuralbiology.cau.edu.cn/PNRD
6.	lncRNAdb v2.0	Quek et al. (2015) Nucleic Acids Research [36]	*A. thaliana* and other plant species such as *O. sativa*, *Medicago truncatula*, *Brassica rapa*, *Glycine max*, etc.	Seven lncRNA entries for *A. thaliana* and single-digit entries for other plant species	Reference database for functional lncRNAs, which have been experimentally validated. Not plant-specific	Manually curated from evidence supported by the literature	http://www.lncrnadb.org/
7.	PLNlncRbase	Xuan et al. (2015) Gene [96]	Forty-three plant species	1187 lncRNAs	A resource for experimentally validated lncRNAs Plant-specific	Manually curated from evidence supported by the literature (over 200 studies)	http://bioinformatics.ahau.edu.cn/PLNlncRbase
8.	GreeNC	Gallart et al. (2016) Nucleic Acids Research [97]	Thirty-seven plant species and 6 algae	>120,000 (high-confidence) lncRNAs	A wiki-based information-rich database of lncRNAs Plant-specific	In silico identification based on data downloaded from Phytozome v10.3	http://greenc.sciencedesigners.com/
9.	CANTATAdb	Szczesśniak et al. (2016) Plant Cell Physiology [98]	10 plant species: *Amborella trichopoda*, *A. thaliana*, *Chlamydomonasreinhardtii*, *G. max*, *O. sativa*, *Physcomitrella patens*, *Selaginellamoellendorffii*, *Solanum tuberosum*, *Vitis vinifera*, and *Z. mays.*	45,117 lncRNAs	A database of lncRNAs with extended annotation like information about lncRNA-miRNA interactions Plant-specific	In silico identification based on publicly available RNA-Seq sample data	http://cantata.amu.edu.pl/
10.	DsTRD	Shao et al. (2016) PLoS ONE [99]	*Salvia miltiorrhiza*	27,687 lncRNAs	A transcriptional resource database specific for the medicinal plant danshen	In silico identification using an in-house Perl script	http://bi.sky.zstu.edu.cn/DsTRD/home.php
11.	RNACentral	The RNAcentral Consortium. (2016) Nucleic Acids Research [100]	*Z. mays* and *A. thaliana*	≈673 lncRNAs	A gateway for the users to access lncRNAs and other ncRNAs via single entry point Not plant-specific	40 expert databases	http://rnacentral.org/
12.	PLncRNAdb	Ming Chen’s Lab [101]	Four plant species: *A. thaliana*, *Arabidospsis lyrata*, *P. trichocarpa*, and *Z. mays*	5000 lncRNAs	A database of lncRNAs with distinct annotation like information lncRNAs and various RBPs Plant-specific	In silico identification and the literature	http://bis.zju.edu.cn/PlncRNADB/index.php

Abbreviations used in Table 2: AFGC, Arabidopsis Functional Genomics Consortium; JGI, Joint Genome Institue; lncRNA, long non-coding RNA; NAT, natural antisense transcript; ncRNA, non-coding RNA; RBP, RNA binding protein.
